# A trio-binned, haplotype-resolved genome sequence of the zebrafish, *Danio rerio *Hamilton 1822, SAT strain

**DOI:** 10.12688/wellcomeopenres.25185.1

**Published:** 2025-12-22

**Authors:** Kerstin Howe, Arang Rhie, Sergey Koren, Elisabeth Busch-Nentwich, Shane A. McCarthy, Jonathan M. D. Wood, Michelle Smith, Gene Myers, Karen Oliver

**Affiliations:** 1 Wellcome Sanger Institute, Hinxton, England, UK; 2 National Human Genome Research Institute (NHGRI), Research Services, Bethesda, Maryland, USA; 3 School of Biological and Behavioural Sciences, Queen Mary University of London, London, England, UK; 4 University of Cambridge Department of Genetics, Cambridge, England, UK; 5 Max Planck Institute of Molecular Cell Biology and Genetics, Dresden, Germany

**Keywords:** Danio rerio SAT strain, zebrafish, trio-binned assembly, haplotype-resolved genome, phased assembly, chromosome-scale, Cypriniformes

## Abstract

We present the trio-binned, haplotype-resolved genome assemblies (released in 2020) of both haplotypes of an individual of the *Danio rerio* SAT strain, a cross between Tuebingen (maternal) and AB (paternal) strains (zebrafish; Chordata; Actinopteri; Cypriniformes; Cyprinidae). The genome sequence of the paternal haplotype (fDreABH1) is 1,354.1 megabases long, while the genome sequence of the maternal haplotype (fDreTuH) is 1,360.5 megabases long. Most of the assembly is scaffolded into 25 chromosomal pseudomolecules. The mitochondrial genome has also been assembled and is 16.6 kilobases in length.

## Species taxonomy

Eukaryota; Opisthokonta; Metazoa; Eumetazoa; Bilateria; Deuterostomia; Chordata; Craniata; Vertebrata; Gnathostomata; Teleostomi; Euteleostomi; Actinopterygii; Actinopteri; Neopterygii; Teleostei; Osteoglossocephalai; Clupeocephala; Otomorpha; Ostariophysi; Otophysi; Cypriniphysae; Cypriniformes; Cyprinoidei; Danionidae; Danioninae; *Danio*; *Danio rerio* (Hamilton, 1822) (NCBI:txid7955).

## Background

*Danio rerio*, commonly known as the zebrafish, serves as a model organism in various fields of biological research, including genetics, developmental biology, and toxicology ( Lieschke & Currie, 2007). Native to the freshwater habitats of South Asia, the genome of this teleost fish was fully sequenced in 2013 ( Howe *et al.*, 2013). The genome comprises approximately 1.4 gigabases, distributed across 25 chromosomes. Notably, the zebrafish genome exhibits a high degree of synteny with the human genome, making it a valuable tool for studying gene function and regulation ( Howe *et al.*, 2013; Postlethwait *et al.*, 2000).

One of the advantages of using *Danio rerio* as a model organism is its rapid embryonic development, which is largely transparent and easily observable under a microscope ( Kimmel *et al.*, 1995). This facilitates real-time analysis of developmental processes. Additionally, the zebrafish is amenable to genetic manipulation, including techniques such as CRISPR/Cas9, which allows for targeted gene editing ( Hwang *et al.*, 2013). Its relatively low maintenance cost and high fecundity further contribute to its utility in research settings ( Lawrence, 2007).

The availability of a fully sequenced genome has accelerated functional genomics studies in zebrafish, enabling researchers to perform genome-wide association studies (GWAS), transcriptomics, and other high-throughput analyses ( Lieschke & Currie, 2007). Consequently, *Danio rerio* continues to be an indispensable resource in advancing our understanding of vertebrate biology, genetics, and disease mechanisms.

In addition to its genomic attributes, the zebrafish model is further enriched by the diversity of its classical laboratory strains. These strains, including AB (ZFIN ID: ZDB-GENO-960809–7), Tuebingen (TU; ZFIN ID: ZDB-GENO-990623–3), and WIK (ZFIN ID: ZDB-GENO-010531–2), have originated from distinct genetic backgrounds. They are a rich source of genetic and phenotypic diversity, which is essential for a broad range of comparative studies ( Brown *et al.*, 2012). This diversity among strains allows for a more comprehensive understanding of genetic influences on various biological processes and phenotypes.

The latest *D. rerio* reference genome is based on the Tuebingen (TU) strain (GCA_000002035.4). A high-quality *de novo* genome assembly of the AB strain of *Danio rerio* has been generated ( Deng *et al.*, 2022), and was compared to the Tuebingen reference genome. Significant structural differences and extensive sequence divergence were identified, including millions of single nucleotide polymorphisms, insertions, deletions, and inversions. These findings highlight the genetic variation within zebrafish strains and underscore the importance of considering these differences in experimental design and comparative studies.

Here we present the genome assembly of an individual of the *Danio rerio* SAT strain (ZFIN ID: ZDB-GENO-100413-1), a cross between Tuebingen and AB, based on a sample provided by Elisabeth Busch-Nentwich, University of Cambridge.

## Genome sequence report

This project provides the genome assemblies of the maternal (Tuebingen) haplotype and the paternal (AB) haplotype of an individual of the *Danio rerio* SAT strain (ZFIN ID: ZDB-GENO-100413-1), based on a sample provided by Elisabeth Busch-Nentwich, University of Cambridge. The assembly of the haplotypes fDreABH1.1 and fDreTuH1.2 is based on 67× PacBio Sequel II data generated by Gene Myers at the MPI of Molecular Cell Biology and Genetics, Dresden, 96× 10X Genomics Chromium data, BioNano data, and 83x Dovetail Hi-C data (from a different individual, SAMEA104026397, fDreSAT3) generated at the Wellcome Sanger Institute. The trioCanu assembly was created by Arang Rhie at NHGRI, and subsequent steps were carried out at the Sanger Institute. The mitochondrial assembly is provided by the Vertebrate Genomes Project team at The Rockefeller University.

**Figure 1.**
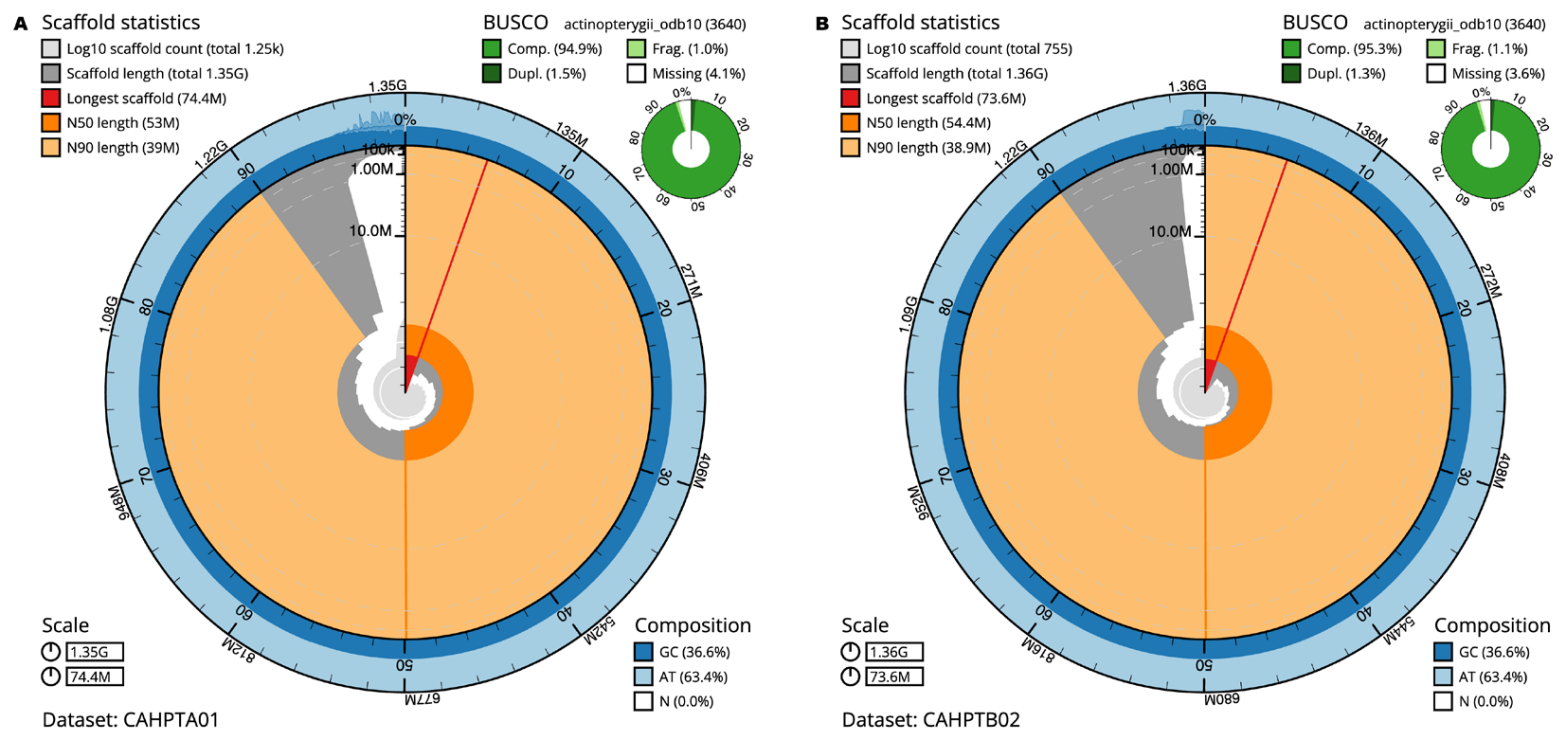
Genome assemblies of *Danio rerio* SAT: metrics. The BlobToolKit snail plots show N50 metrics and BUSCO gene completeness. The main plot is divided into 1,000 size-ordered bins around the circumference with each bin representing 0.1% of the assembly. The distribution of sequence lengths is shown in dark grey with the plot radius scaled to the longest sequence present in the assembly (shown in red). Orange and pale-orange arcs show the N50 and N90 sequence lengths, respectively. The pale grey spiral shows the cumulative sequence count on a log scale with white scale lines showing successive orders of magnitude. The blue and pale-blue area around the outside of the plot shows the distribution of GC, AT and N percentages in the same bins as the inner plot. A summary of complete, fragmented, duplicated and missing BUSCO genes in the actinopterygii_odb10 set is shown in the top right. The snail plots can be viewed interactively at the following links: fDreABH1 and for fDreTuH1.

**Figure 2.**
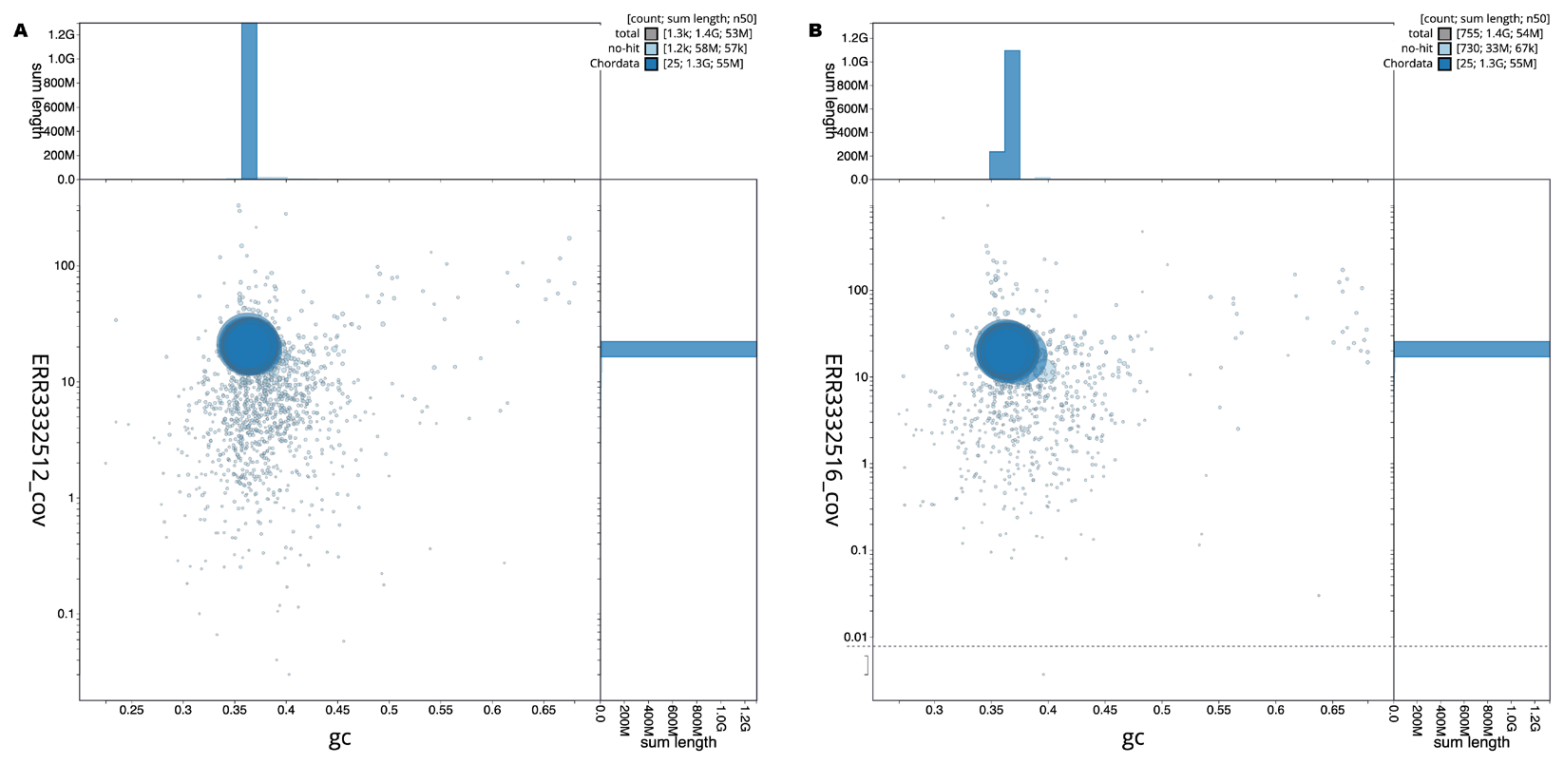
Genome assemblies of *Danio rerio* SAT: BlobToolKit GC-coverage plot. Scaffolds are coloured by phylum. Circles are sized in proportion to scaffold length. Histograms show the distribution of scaffold length sum along each axis. The blob plots can be viewed interactively at the following links: fDreABH1 and for fDreTuH1.

The final fDreABH1.1 assembly has a total length of 1,354.1 Mb in 1,252 sequence scaffolds with a scaffold N50 of 53.0 Mb ( Table 1). The final fDreTuH1.2 assembly has a total length of 1,360.5 Mb in 754 sequence scaffolds with a scaffold N50 of 54.4 Mb ( Table 1). The snail plots in Figure 1 provide a summary of the assembly statistics, while the distribution of assembly scaffolds on GC proportion and coverage is shown in Figure 2.

**Table 1.** Genome data for *Danio rerio*, fDreTuH1.2.

Project accession data	Paternal	Maternal
Assembly identifiers	fDreABH1.1	fDreTuH1.2
Species	*Danio rerio*	
Specimen	fDreABH1	fDreTuH1
NCBI taxonomy ID	7955	
BioProject	PRJEB38590	
BioSample ID	SAMEA4760846	SAMEA4760849
Assembly metrics *	fDreABH1.1	fDreTuH1.2
BUSCO *	C:94.9%[S:93.3%,D:1.5%], F:1.0%,M:4.1%,n:3,640	C:95.3%[S:94.0%,D:1.3%], F:1.1%,M:3.6%,n:3,640
Mitochondrial assembly	-	LR822066.1 16.6 kb
Raw data accessions		
10X Genomics Illumina	ERR3332513, ERR3332515, ERR3332516, ERR3332517, ERR3332514, ERR3332511, ERR3332512, ERR3332510,	
Hi-C-dovetail	ERR4021773	
Genome assembly		
Assembly accession	GCA_903684865.1	GCA_903684855.2
Span (Mb)	1,354.1	1,360.5
Number of contigs	1,964	2,104
Contig N50 length (Mb)	4.5	3.6
Number of scaffolds	1,252	754
Scaffold N50 length (Mb)	53.0	54.4
Longest scaffold (Mb)	74.43	73.59

* BUSCO scores based on the actinopterygii_odb10 BUSCO set using v5.3.2. C = complete [S = single copy, D = duplicated], F = fragmented, M = missing, n = number of orthologues in comparison. Full sets of BUSCO scores are available for fDreABH1 and fDreTuH1.2.

**Figure 3.**
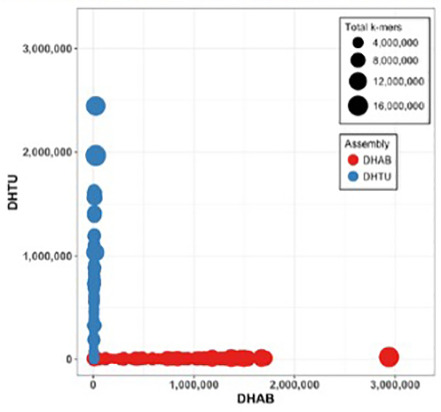
Haplotype *k*-mer validation for the SAT trio-binned assembly. Each circle represents a contig, with size proportional to total *k*-mer content. The *x*-axis shows AB-specific k-mer counts (DHAB: double haploid AB parent), and the *y*-axis shows Tuebingen-specific k-mer counts (DHTU: double haploid Tuebingen parent). Red circles: contigs assigned to the fDreABH1 (paternal) assembly; blue circles: contigs assigned to the fDreTuH1 (maternal) assembly. Successful phasing is demonstrated by contigs clustering near their respective axes.

The trio binning approach successfully separated the parental haplotypes based on *k-*mer classification. Haplotype-specific *k*-mer analysis confirmed that contigs were correctly assigned to their respective parental haplotypes, with minimal cross-contamination between the DHAB (paternal) and DHTU (maternal) assemblies ( Figure 3).

**Figure 4.**
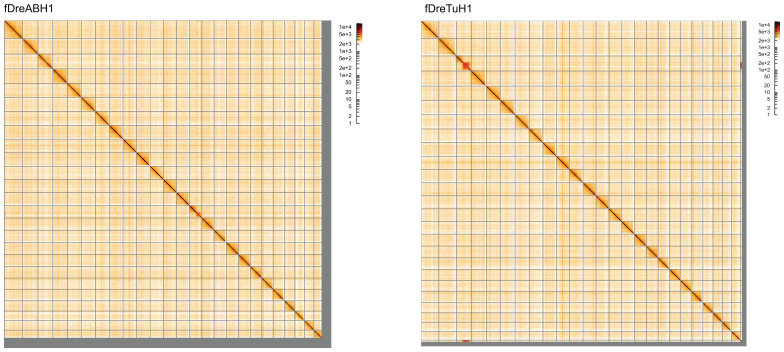
Genome assemblies of *Danio rerio* SAT: Hi-C contact map of the assemblies, visualised using HiGlass. Chromosomes are shown in order of size from left to right and top to bottom. The panel on the left shows the fDreABH1 assembly and the right show the fDreTuH1 assembly. The maps can be viewed interactively at the following links: fDreABH1 and for fDreTuH1.

Most (95.69%) of the assembly sequence was assigned to 25 chromosomal-level scaffolds. Chromosome-scale scaffolds confirmed by the Hi-C data are named by synteny ( Figure 4; Table 2). The mitochondrial genome was also assembled and can be found as a contig within the multifasta file of the genome submission.

**Table 2.** Chromosomal pseudomolecules in the genome assembly of *Danio rerio*, fDreSAT1.

	fDreABH1 (paternal haplotype)		fDreTuH1.2 (maternal haplotype)	
Chromosome	INSDC accession	Length (Mb)	INSDC accession	Length (Mb)
1	LR812038.1	59.96	LR812063.1	59.57
2	LR812039.1	57.65	LR812064.1	59.97
3	LR812040.1	62.6	LR812065.1	62.13
4	LR812041.1	51.23	LR812066.1	62.64
5	LR812042.1	74.43	LR812067.1	72.58
6	LR812043.1	59.69	LR812068.1	59.35
7	LR812044.1	32.28	LR812069.1	73.59
8	LR812045.1	55.94	LR812070.1	55.32
9	LR812046.1	57.07	LR812071.1	56.85
10	LR812047.1	46.41	LR812072.1	45.64
11	LR812048.1	46.06	LR812073.1	45.93
12	LR812049.1	49.74	LR812074.1	36.19
13	LR812050.1	52.96	LR812075.1	53.21
14	LR812051.1	54.75	LR812076.1	53.9
15	LR812052.1	50.01	LR812077.1	49.37
16	LR812053.1	55.93	LR812078.1	55.35
17	LR812054.1	54.64	LR812079.1	54.39
18	LR812055.1	52.5	LR812080.1	52.04
19	LR812056.1	49.85	LR812081.1	49.53
20	LR812057.1	56.54	LR812082.1	55.94
21	LR812058.1	48.05	LR812083.1	47.18
22	LR812059.1	39.02	LR812084.1	38.89
23	LR812061.1	47.47	LR812085.1	47.27
24	LR812061.1	42.17	LR812086.1	42.36
25	LR812062.1	38.62	LR812087.1	38.18
MT	-	-	LR822066.1	0.02

For the maternal haplotype (fDreTuH1.2), the estimated Quality Value (QV) of the final assembly is 35.5 with *k*-mer completeness of 99.13%, and the assembly has a BUSCO completeness of 95.3% (single = 94.0%, duplicated = 1.3%). BUSCO version 5.3.2 was used, using the actinopterygii_odb10 reference set ( *n* = 3,640). For the paternal haplotype (fDreABH1.1), the estimated Quality Value (QV) of the final assembly is 36.4 with *k*-mer completeness of 99.31%, and the assembly has a BUSCO completeness of 94.9% (single = 93.3%, duplicated = 1.5%).

## Methods

### Sample acquisition and nucleic acid extraction

A sample of an individual of the *Danio rerio* SAT strain was provided by Elisabeth Busch-Nentwich, University of Cambridge. This strain is a cross between a female of the Tuebingen haplotype and a male of the AB haplotype.

Nucleic acid extraction was carried out using Bionano Prep Cell Culture DNA Isolation Protocol. In this method, the cells are first embedded in agarose to provide structural support during the extraction process. The agarose-embedded cells are then treated with lysis buffers to break down the cell membranes and release the DNA. The process also involves proteinase digestion to remove proteins, followed by a series of washes to purify the DNA.

### Sequencing

Pacific Biosciences CLR (continuous long reads) and 10X Genomics read cloud DNA sequencing libraries were constructed according to the manufacturers’ instructions. DNA sequencing was performed by the Scientific Operations core at the WSI on Pacific Biosciences SEQUEL II (CLR) and Illumina HiSeq X Ten instruments. Additionally, BioNano optical maps were generated to provide high-resolution genome mapping. Discovar sequencing of the maternal and paternal samples was conducted on Illumina platforms to improve genome assembly accuracy. Hi-C data were also generated using the Dovetail Hi-C kit and sequenced on the HiSeq X Ten instrument.

### Genome assembly, curation and evaluation

The fDreABH1.1 and fDreTuH1.2 haplotype assemblies are based on 67× PacBio CLR data generated by Gene Myers at the MPI of Molecular Cell Biology and Genetics, Dresden, 96× 10X Genomics Chromium data, BioNano data, and 83x Dovetail Hi-C data (from a different individual, SAMEA104026397, fDreSAT3) generated at the Wellcome Sanger Institute. The assembly process included the following sequence of steps: segregation of PacBio reads representing the two haplotypes using trioCanu ( Koren *et al.*, 2018) and *k*-mers from the Illumina-sequenced parents (maternal: SAMEA3891248 at 143× coverage, and paternal: SAMEA3891249 at 136× coverage), separate assembly of each haplotype using Canu ( Koren *et al.*, 2017), polishing with Arrow from the binned reads, retained duplication removal with purge_dups, 10X based scaffolding with scaff10x, BioNano hybrid-scaffolding, Hi-C based scaffolding with SALSA2 ( Ghurye *et al.*, 2019), further Arrow polishing, and two rounds of FreeBayes ( Garrison & Marth, 2012) Illumina polishing. The mitochondrial assembly was produced by the Vertebrate Genomes Laboratory at Rockefeller University using mitoVGP. Finally, the assembly was analysed and manually improved using gEVAL ( Chow *et al.*, 2016), and marker placement from the high-density genetic map SATmap ( Howe *et al.*, 2013) to achieve chromosomal resolution ( Table 3).

**Table 3.** SATMAP marker alignment to haplotype assemblies. Using exonerate to align 140,000 SATMAP markers to individual haplotype assemblies, counting markers that map 100% and only once.

SATMAP aligns unique	DHAB marker	DHTU marker
**DHAB**	121,208	14,882
**DHTU**	14,975	126,824

To evaluate the final genome assemblies, the genome was analysed within the BlobToolKit environment ( Challis *et al.*, 2020) and BUSCO scores ( Manni *et al.*, 2021) were calculated. Hi-C maps were produced using bwa-mem2 ( Vasimuddin *et al.*, 2019) in the Cooler file format ( Abdennur & Mirny, 2020).

Alignment of parental Illumina reads to the haplotype assemblies using minimap2 confirmed the correct assignment of sequences, with 97.55% of AB reads mapping to the fDreABH1 assembly and 95.4% of Tuebingen reads mapping to the fDreTuH1 assembly ( Table 4).

**Table 4.** Parental read alignment validation. Alignment of parental reads to the concatenated haplotype assemblies using minimap2, showing percentage of reads with MAPQ>30 mapping to each assembly.

	AB reads	Tuebingen reads
**Mapped to fDreABH1**	97.55%	4.59%
**Mapped to fDreTuH1**	2.44%	95.4%

### Wellcome Sanger Institute – Legal and Governance

The materials that have contributed to this genome note have been supplied by a Tree of Life collaborator. The Wellcome Sanger Institute employs a process whereby due diligence is carried out proportionate to the nature of the materials themselves, and the circumstances under which they have been/are to be collected and provided for use. The purpose of this is to address and mitigate any potential legal and/or ethical implications of receipt and use of the materials as part of the research project, and to ensure that in doing so we align with best practice wherever possible.

The overarching areas of consideration are:

•   Ethical review of provenance and sourcing of the material

•   Legality of collection, transfer and use (national and international)

Each transfer of samples is undertaken according to a Research Collaboration Agreement or Material Transfer Agreement entered into by the Tree of Life collaborator, Genome Research Limited (operating as the Wellcome Sanger Institute) and in some circumstances other Tree of Life collaborators.

## Data Availability

European Nucleotide Archive: *Danio rerio* SAT strain (zebrafish) haplotype-resolved. Accession number PRJEB38590; https://identifiers.org/ena.embl/PRJEB38590. The genome sequence is released openly for reuse. The *Danio rerio* genome sequencing initiative is part of the Vertebrate Genomes Project. All raw sequence data and the assembly have been deposited in INSDC databases. Raw data and assembly accession identifiers are reported in Table 1.
