# Supplementary material for: A trio-binned, haplotype-resolved genome sequence of the zebrafish, Danio rerio Hamilton 1822, SAT strain
Source: Wellcome Open Res. 2025 Dec 22;10:682. [Version 1] doi: 10.12688/wellcomeopenres.25185.1 (PMC12954400; doi:10.12688/wellcomeopenres.25185.1)
Supplement: Peer review history

# Reviewer report: Reviewer response for version 1

**DOI:** 10.21956/wellcomeopenres.27760.r143732

**Published:** 2026-03-02

**Authors:** Annabel Whibley

**Affiliations:** 1 The University of Auckland, Auckland, New Zealand; 2 Grapevine Improvement, Bragato Research Institute, Lincoln, New Zealand

Howe and colleagues report the trio-binned assembly of the SAT strain of *Danio rerio*, an important model species. The SAT strain is a cross between the Tuebingen (TU) strain, which constitutes the original *D. rerio* reference background, and another classical laboratory strain known as AB. The abstract notes that the assemblies were released in 2020- it is not clear to me whether the motivation for this report was to bring the assembly evaluation in line with other zebrafish resources, or simply to provide framing information to a long-available assembly dataset, a clarifying statement to explain the motivation might be helpful.

 It is presumably considered beyond the scope of a Data Note to undertake extensive comparative analyses, but I remain curious about this, including how the assembly relates to other attempts to assemble the same strain, such as that reported by  Chernyavskaya Y, Zhang X, Liu J, Blackburn J. Long-read sequencing of the zebrafish genome reorganizes genomic architecture. BMC Genomics. 2022 Feb 10;23(1):116. doi: 10.1186/s12864-022-08349-3.

 The maternal and paternal haplotypes have comparable properties (contig number, completeness, scaffold N50). Kmer-validation of the trio-binning, using doubled-haploid parental confirmation looks very clean. The assemblies were released in 2020 and the cocktail of sequencing technologies used reflects the state of the field at the time, e.g. using PacBio CLR, Chromium 10x. I think this is reflected in the QV value of ~35, which would generally be bettered with present-day sequencing datasets, but aside from this the quality parameters of the assembly appear on a par with current high quality references.  The assembly process is more complex and convoluted than the present-day DToL pipeline and there would be some value to setting out technical aspects and parameters of the assembly pipeline more clearly. “Discovar” sequencing is mentioned in the Sequencing section and I think would benefit from clarification/referencing (presumably this means the generation of overlapping PE Illumina reads to obtain ~250bp contigs?).

 Minor additional comments:

 Consider removing the redundancy between the final sentence of the background and the opening of the Genome sequence report section (and also the opening sentence of the Methods).  

 Accession identifier for Chromosome 23 for fDreABH1 should be LR812060.1 not  LR812061.1

Are sufficient details of methods and materials provided to allow replication by others?

Partly

Is the rationale for creating the dataset(s) clearly described?

Yes

Are the datasets clearly presented in a useable and accessible format?

Yes

Are the protocols appropriate and is the work technically sound?

Yes

Reviewer Expertise:

Bioinformatics, Genomics

I confirm that I have read this submission and believe that I have an appropriate level of expertise to confirm that it is of an acceptable scientific standard.

# Reviewer report: Reviewer response for version 1

**DOI:** 10.21956/wellcomeopenres.27760.r143157

**Published:** 2026-01-21

**Authors:** Tim Martin Knutsen

**Affiliations:** 1 AquaGen AS, Trondheim, Norway

**Summary of the manuscript**

 The authors present a trio-binned, haplotype-resolved genome assembly of a zebrafish ( *Danio rerio*) SAT strain individual, which represents a cross between the Tuebingen (maternal) and AB (paternal) laboratory strains . Using PacBio long reads from the offspring and short-read data from both parents, the authors generate separate haplotype assemblies that are scaffolded to chromosome scale and validated using k-mer analysis, parental read mapping, BUSCO completeness, and Hi-C data . The resulting assemblies are deposited in public archives and intended as a resource for the zebrafish community.

**Context and significance**

 Zebrafish is one of the most widely used vertebrate model organisms, and substantial genetic divergence between common laboratory strains such as AB and Tuebingen is well documented. In this context, haplotype-resolved assemblies derived from an F1 cross have clear potential value for reducing reference bias, enabling strain-aware analyses, and improving structural variant interpretation.

 However, the manuscript would benefit from placing this dataset more clearly in the context of existing zebrafish genomic resources. While the authors note that the assemblies were released in 2020, the Data Note was not published until December 2025, representing a five-year delay . For a Data Note, this is not necessarily problematic, but the authors should explicitly clarify what this publication adds beyond the original release. 

 In addition, unlike several other trio-binning genome reports, the introduction does not clearly explain why trio binning was chosen, nor does it explicitly highlight that the sequenced individual represents an F1 cross rather than offspring from two SAT-strain parents. Adding a short, method-focused explanation early in the manuscript would make the work more accessible and better contextualized.

**Major and minor comments**

 Genome sequence report and abstract

- Inconsistent assembly identifiers:
  In the abstract, the haplotypes are referred to as *fDreABH1* and *fDreTuH*, whereas later in the manuscript the versioned identifiers *fDreABH1.1* and *fDreTuH1.2* are used consistently (for example in the Genome sequence report and Table 1) . These identifiers should be standardized throughout the manuscript to avoid confusion.
- Introduction could better explain trio binning:
  The background section provides a general overview of zebrafish biology and laboratory strains, but it would benefit from a brief introduction to trio-binned assembly approaches, explaining how parental short reads are used to partition offspring long reads into maternal and paternal bins, and why this improves haplotype resolution. This is particularly important here, as the key strength of the dataset lies in the phased nature of the assemblies.
- Clarification of the F1 design:
  Although the Methods state that the SAT individual is a cross between a female Tuebingen and male AB haplotype , this fact is not clearly highlighted in the introduction. Explicitly stating that this is an F1 cross used for trio binning, rather than a generic SAT-strain individual, would improve clarity.

Figures

- Figure quality and interpretability:
  The figures in the Genome sequence report (BlobToolKit snail plots, GC-coverage plots, and Hi-C maps) contain dense information and small labels. In the PDF version, several elements are difficult to read without relying on the interactive versions . Providing higher-resolution embedded figures or simplifying figure layouts would improve accessibility for readers who rely primarily on the PDF.
- BUSCO interpretation across haplotypes:
  BUSCO results are reported in the text and Table 1, but the figures do not clearly facilitate direct comparison between haplotypes. Given that haplotype resolution is a central contribution, a short explanatory sentence in the text clarifying why both haplotypes show similar BUSCO completeness and how duplicated BUSCOs should be interpreted would strengthen the presentation.

Methods

- Need for a more complete software and version table:
  While the manuscript lists the tools used (for example trioCanu, Canu, SALSA2, FreeBayes, gEVAL), software versions are scattered throughout the text or absent for some steps . A concise table summarizing all software, versions, and key parameters would greatly improve reproducibility and align with best practices for Data Notes.
- Sample collection and handling:
  The sample acquisition section states that material was provided by a collaborator and describes DNA extraction in general terms . The manuscript would benefit from slightly more detail on sample provenance and handling (for example tissue type, developmental stage, storage conditions), even if standard, to support reproducibility and transparency.
- Use of Hi-C data from a different individual:
  Hi-C data used for scaffolding were generated from a different individual (SAMEA104026397) . While this is not uncommon, a brief justification and statement on potential implications for haplotype scaffolding would be useful.

Data presentation

- Potential accession error in Table 2:
  In Table 2, the paternal haplotype accession LR812061.1 appears to be listed for both chromosome 23 and chromosome 24 . This looks like a duplication error and should be checked and corrected.

Are sufficient details of methods and materials provided to allow replication by others?

Partly

Is the rationale for creating the dataset(s) clearly described?

Yes

Are the datasets clearly presented in a useable and accessible format?

Partly

Are the protocols appropriate and is the work technically sound?

Yes

Reviewer Expertise:

Bioinformatics, genome assembly, variant calling.

I confirm that I have read this submission and believe that I have an appropriate level of expertise to confirm that it is of an acceptable scientific standard, however I have significant reservations, as outlined above.

# Reviewer report: Reviewer response for version 1

**DOI:** 10.21956/wellcomeopenres.27760.r143733

**Published:** 2026-01-20

**Authors:** Jayan Duminda M Senevirathna

**Affiliations:** 1 Uva Wellassa University, Badulla, Uva Province, Sri Lanka

The authors report BUSCO completeness values of approximately 95% for both haplotype assemblies, indicating that each haplotype captures the majority of expected conserved genes. However, this important result is not clearly or consistently presented in Figures though noted as A and B (e.g., figure 1). The figures do not explicitly distinguish BUSCO completeness between haplotype 1 and haplotype 2, nor does it clearly indicate whether the reported BUSCO scores refer to complete, duplicated, fragmented, or missing categories for each haplotype.

 Given that haplotype-resolved assemblies are a key strength of the study, the lack of clarity in Figures limits the reader’s ability to properly evaluate assembly quality and phasing performance. The authors are encouraged to revise Figures to clearly label BUSCO results for each haplotype separately (e.g., stacked bars or side-by-side comparisons), and to ensure consistency between the figure, figure legend, and text.

 Additionally, the manuscript would benefit from a brief discussion explaining why both haplotypes show similar (~95%) BUSCO completeness, including whether duplicated BUSCOs were expected and how this reflects heterozygosity and phasing accuracy. Clarifying these points would significantly improve transparency and strengthen the interpretation of the haplotype-resolved genome assembly.

Are sufficient details of methods and materials provided to allow replication by others?

Yes

Is the rationale for creating the dataset(s) clearly described?

Yes

Are the datasets clearly presented in a useable and accessible format?

Yes

Are the protocols appropriate and is the work technically sound?

Yes

Reviewer Expertise:

Aquatic Biotechnology and Genetics

I confirm that I have read this submission and believe that I have an appropriate level of expertise to confirm that it is of an acceptable scientific standard, however I have significant reservations, as outlined above.
